# Nutritional supplements and mother’s milk composition: a systematic review of interventional studies

**DOI:** 10.1186/s13006-020-00354-0

**Published:** 2021-01-04

**Authors:** Mojtaba Keikha, Ramin Shayan-Moghadam, Maryam Bahreynian, Roya Kelishadi

**Affiliations:** 1 Department of Public Health, Sirjan School of Medical Sciences, Sirjan, Iran; 2 Student Research Committee, School of Medicine, Isfahan University of Medical Sciences, Isfahan, Iran; 3 Department of Nutrition, Child Growth and Development Research Center, Research Institute for Primordial Prevention of Non-communicable Disease, Isfahan University of Medical Sciences, Isfahan, Iran; 4 Department of Pediatrics, Child Growth and Development Research Center, Research Institute for Primordial Prevention of Non-communicable Disease, Isfahan University of Medical Sciences, Isfahan, Iran

**Keywords:** Dietary supplements, Human milk, Vitamins, Minerals, Breast-milk composition

## Abstract

**Background:**

This study aims to systematically review the effects of maternal vitamin and/or mineral supplementation on the content of breast milk.

**Methods:**

We systematically searched electronic databases including Medline via PubMed, Scopus and ISI Web of Science till May 24, 2018. The following terms were used systematically in all mentioned databases: (“human milk” OR “breast milk” OR “breast milk composition” OR “human breast milk composition” OR “composition breast milk” OR “mother milk” OR “human breast milk” OR “maternal milk”) AND (“vitamin a” OR “retinol” OR “retinal” OR “retinoic acid” OR “beta-carotene” OR “beta carotene” OR “ascorbic acid” OR “l-ascorbic acid” OR “l ascorbic acid” OR “vitamin c” OR “vitamin d” OR “cholecalciferol” OR “ergocalciferol” OR “calciferol” OR “vitamin e” OR “tocopherol” OR “tocotrienol” OR “alpha-tocopherol” OR “alpha tocopherol” OR “α-tocopherol” OR “α tocopherol” OR “vitamin k” OR “vitamin b” OR “vitamin b complex” OR “zinc” OR “iron” OR “copper” Or “selenium” OR “manganese” OR “magnesium”) and we searched Medline via Medical subject Headings (MeSH) terms. We searched Google Scholar for to increase the sensitivity of our search. The search was conducted on human studies, but it was not limited to the title and abstract. Methodological quality and risk of bias of included studies were evaluated by Jadad scale and Cochrane risk of bias tools, respectively.

**Results:**

This review included papers on three minerals (zinc, iron, selenium) and 6 vitamins (vitamin A, B, D, C, E and K) in addition to multi-vitamin supplements. Although studies had different designs, e.g. not using random allocation and/or blinding, our findings suggest that maternal use of some dietary supplements, including vitamin A, D, vitamin B1, B2 and vitamin C might be reflected in human milk. Vitamin supplements had agreater effect on breast milk composition compared to minerals. Higher doses of supplements showed higher effects and they were reflected more in colostrum than in the mature milk.

**Conclusion:**

Maternal dietary vitamin and/or mineral supplementation, particularly fat- soluble vitamins, vitamin B1, B2 and C might be reflected in the breast milk composition. No difference was found between mega dose and single dose administration of minerals.

## Background

Human milk is known as the most convenient and available food source for infants in the first 6 months of life. Consuming breast milk should be continued until the end of the second year of an infant’s life with suitable complementary foods. Human milk has a substantial effect on infant growth and development [1, 2]. Consequently, study on the composition of human milk is of crucial importance.

Maternal breast milk composition usually depends on maternal nutrition [3, 4], as has been indicated in previous studies [2–5]. A systematic review found that maternal dietary intake, particularly fatty acids, and some micronutrients, such as fat-soluble vitamins, vitamin B1 and C, was associated with micronutrient content in breast milk [2].

Micronutrients and vitamins are also very important factors for the development and growth of infants. Micronutrients and vitamins have a profound effect on the neural development of children, metabolic processes, development of soft tissues and muscles, transport of oxygen and synthesis of DNA. Micronutrients and vitamins also have anti-infectious effects and anti-oxidant effect, which is very important in infancy [3, 4]. Lack of some of micronutrients and vitamins cause some diseases such as rickets and vitamin d, hemolytic anemia and also severe anemia with iron, hydrocephalus and vitamin B12, xerophthalmia and vitamin A and recurrent infectious diseases with vitamin A and E [6, 7].

Many studies have been done on the composition of human milk and comparing human milk composition with infant formula. Unlike infant formula which has a standard and fixed composition, human milk composition varies due to factors such as maternal age, maternal parity, nutritional factors, behavioral factors, maternal hormones, environmental factors, infant sex, time of lactation and many other factors [8, 9]. One of the biggest impacts on human milk composition is maternal nutritional status and diet. Many studies have investigated the effect of the amount and type of foods and supplements, such as micronutrients and vitamins that were consumed by mothers, and their effect on human milk. The results of these surveys were widely varied and occasionally contrasting. Maternal diet can influence her milk combination by different metabolic pathways that produce indirect effects and also some metabolic pathways regulate certain human milk combination directly through dietary intake [9–11].

In our recently published systematic review by on the effect of maternal diet on human milk composition [2], we found that the association for some elements were stronger including fatty acids and an attenuated association was found with fat soluble vitamins, vitamin B1, and vitamin C. The effects of maternal nutrition on breast milk composition are not the same in all components of macro- and micronutrients; therefore, the question is raised whether maternal supplement use can affect milk composition more than maternal diet.

Another study conducted on Nigerian mothers and their infants at birth and 6 month, showed that concentrations of fatty acids and vitamins such as vitamins A, C, and B6 reflected the respective dietary intakes of these nutrients in the maternal diet [12]. Conversely some other studies showed that the mineral content of human milk is generally considered less related to maternal dietary intakes [13–15].

Previous results on the effect of maternal micronutrients and vitamin intake on human milk are contradictory. Therefore, this study aims to systematically review the effects of maternal vitamin and/or mineral supplementation on breast milk content.

## Methods

The review study was designed in accordance with the protocols of Systematic Review and Meta-Analysis (PRISMA) [16]. The study protocol is registered in the PROSPERO with identification number of CRD42020209008.

### Literature search

We systematically searched the electronic databases including Medline via PubMed, Scopus and ISI Web of Science till May 24, 2018. The following key words were used systematically in all mentioned databases: (“human milk” OR “breast milk” OR “breast milk composition” OR “human breast milk composition” OR “composition breast milk” OR “mother milk” OR “human breast milk” OR “maternal milk”) AND (“vitamin a” OR “retinol” OR “retinal” OR “retinoic acid” OR “beta-carotene” OR “beta carotene” OR “ascorbic acid” OR “l-ascorbic acid” OR “l ascorbic acid” OR “vitamin c” OR “vitamin d” OR “cholecalciferol” OR “ergocalciferol” OR “calciferol” OR “vitamin e” OR “tocopherol” OR “tocotrienol” OR “alpha-tocopherol” OR “alpha tocopherol” OR “α-tocopherol” OR “α tocopherol” OR “vitamin k” OR “vitamin b” OR “thiamin” OR “vitamin B1” OR “vitamin B12” OR “vitamin B6” OR “vitamin B7” OR “vitamin B3” OR “vitamin B2” OR “vitamin b complex” OR “thiamine” OR “riboflavin” OR “niacin” OR “pantothenic acid” OR “pyridoxine” OR “biotin” OR “folate” OR “cobalamin” OR “zinc” OR “iron” OR “copper” OR “selenium” OR “manganese” OR “magnesium”).

Furthermore, we searched Medline via Medical subject Headings (MeSH) terms with following MeSH terms: (“Milk, Human”[Mesh]) AND (“Vitamin A”[Mesh] OR “Ascorbic Acid”[Mesh] OR “Vitamin D”[Mesh] OR “Vitamin E”[Mesh] OR “Vitamin K”[Mesh] OR “Vitamin B Complex”[Mesh] OR “Vitamin K 3”[Mesh] OR “Vitamin K 2”[Mesh] OR “Vitamin K 1”[Mesh] OR “Vitamin B 12”[Mesh] OR “Vitamin B 6”[Mesh] OR “Zinc”[Mesh] OR “Iron”[Mesh] OR “Copper”[Mesh] OR “Selenium”[Mesh] OR “Manganese”[Mesh] OR “Magnesium”[Mesh] OR “Thiamine”[Mesh] OR “Riboflavin”[Mesh] OR “Niacin”[Mesh] OR “Pantothenic Acid”[Mesh] OR “Pyridoxine”[Mesh] OR “Biotin”[Mesh] OR “Folic Acid”[Mesh] OR “Vitamin B 12”[Mesh]).

Also, we searched Google scholar to increase the sensitivity of our search. The search was conducted on human studies, but it was not limited to title and abstract because our desired results or outcomes might have been considered a secondary aim of the studies and mentioned in the full text of articles. Limitations were applied to exclude conference papers, editorials, letters, commentary, short surveys, and notes. We did not consider any time limitation. Only English papers were used in the current review.

### Hand searching

We checked the reference list of the published studies to increase the sensitivity and to identify more related studies.

### Ethical consideration

Ethical approval was not required as this was a secondary study.

### Data management

We used EndNote program (version 6) for managing and handling extracted references that were searched from databases. Duplicates were removed and entered into a duplicate library.

### Selection criteria

Studies identified from the literature search were selected on the basis of the predefined selection criteria presented later:

#### Inclusion criteria

1. All interventional studies (randomized controlled trial, quasi experimental)
2. Studies that have studied the effect of any nutrient (macro or micro) and/or (?) supplements on human milk composition.
3. Studies assessing the composition of mother’s milk.

#### Exclusion criteria

1. Conference papers, editorials, letters, commentary, short surveys, and notes
2. Animal studies
3. Laboratory studies
4. Studies that have studied the effect of any nutrient intake (macro or micro) and/or supplements on blood serum of mothers or infants (only the effect of nutritional supplements on milk composition were included).
5. Studies that used fortified foods, vegetables and/or fruits (oranges, carrots, etc.) rather than dietary supplements.

### Assessment of study quality

For quality assessment we used Jadad scale for classification and ranking the methodological quality of eligible studies [17]. According to the study design such as randomization and blinding, each study was classified with a score ranging from 0 to 5, and studies with Jadad score above 3 were considered a high quality study.

### Quality of included studies and risk of bias assessment

**Fig. 1:**
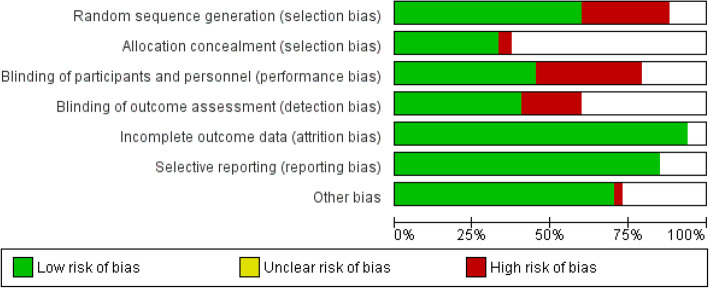
Risk of bias graph review: authors’ judgements on each risk of bias item presented as a percentage for each included study

**Fig. 2:**
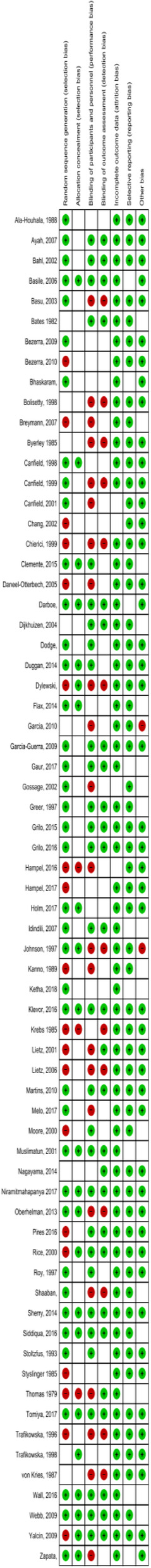
Risk of bias summary review: authors’ judgements on each risk of bias item for each included study

Quality assessment of each included study according to Jadad scale is demonstrated in the last column of Tables 1, 2, 3, 4, 5, 6, 7, 8, 9 and 10. Also, Figs. 1 and 2 show risk of bias item presented as a percentage and risk of bias item for each included study, respectively.

**Table 1:** Summary effect of nutritional vitamins supplements on status of vitamin A human milk composition

First author surname, citation number	Type of Supplement	Characteristics of Participants	Type of study	Aim	Type of Nutrients Evaluated in Milk	Main findings	Jadad scale points and WHO divisions
Ayah [18]	maternal vitamin A (400 000 IU) or placebo,24 h postpartum	435 mothers infant pairs.	A randomised, placebo-controlled, double blind, two-by-two factorial trial	To assess the effects of high-dose postpartum maternal supplementation with 400,000 IU and infant supplementation with 100,000 IU at 14 weeks of age, on maternal and infant vitamin A status in the 6-month postpartum period.	Retinol	Maternal serum retinol was not different between groups, but milk retinol was higher in the vitamin A group. Vitamin A supplementation was associated with significantly higher milk retinol per volume at 4, 14 and 26 weeks postpartum and higher milk retinol expressed per gram fat at week 4, but not at weeks 14 and 26.	5/5 Africa (AFRO)
Bahl [19]	Single dose of 60 mg vitamin A or placebo at enrolment.	A total of 9424 mother-infant pairs were enrolled in the trial, 18–28 d postpartum in India and Peru and 21–42 d after delivery in Ghana.	Multicenter randomized, double-blind, placebo-controlled trial	To determine the effect of maternal vitamin A supplementation on breast milk retinol and of maternal and infant supplementation on infant vitamin A status.	Retinol	Maternal supplementation resulted in higher breast milk retinol at 2 mo postpartum. At 6 and 9 mo, maternal supplementation did not affect breast milk retinol or the proportion of mothers with low breast milk retinol. At the doses used, maternal supplementation improved breast milk retinol status at 2 mo (*P* = 0.001) and maternal and infant supplementation modestly increased (*P* = 0.03) infant vitamin A status at 6 mo of age.	3/5 (SEARO), (AMRO), (AFRO)
Basu [20]	A single oral dose of 209 μmol of retinol (200,000 IU of vitamin A)	300 mothers, 150 in control and 150 in treatment group. Mean age: 24.6 and 25.2 years respectively.	A randomised controlled prospective study	To evaluate the effect of a single oral mega dose of vitamin A on the breast milk concentration.	Retinol	After supplementation, the treatment group showed a rise in mean breast milk retinol content (12.08 *v* 2.96 μmol/l) which remained significantly higher for four months. The breast milk retinol concentration increased significantly in the test population within 24 h of supplementation.	1/5 South East Asia (SEARO)
Bezerra [21]	retinyl palmitate consisted of a single dose of 200,000 IU (experimental group) and zero IU (control group).	113 healthy women aged 18–40 years.	randomised clinical trial	To evaluate the effect of maternal supplementation with a single dose of retinyl palmitate during the postpartum period, in order to supply vitamin A to the infant at the concentration of retinol in maternal milk.	Retinol	There was a significant increase in mean retinol levels in the colostrums of the supplemented group: 3.22 (sd = 1.81) μmol/l and 5.76 (sd = 2.80) μmol/l (*p* < 0.0001), between time zero and 24 h, respectively. This increase did not occur in the control group (*p* = 0.69).	2/5 Americas (AMRO)
Bezerra [22]	Participants were randomly allocated to 3 groups and supplemented in the postpartum period with a single retinyl palmitate dose of 200,000 IU (S1), a double dose of 200,000 IU 24 h apart (S2), or no supplementation (C).	199 healthy women within 16 h postpartum, aged between 18 and 40 years.	randomised clinical trial	To assess the effect of 2 different mega doses of retinyl palmitate on the level of retinol in the breast milk of healthy women.	retinol	The retinol content in mature milk differed between no supplementation group and groups S1 and S2 (*P* < .05). The double dose of vitamin A did not significantly increase the retinol content of milk at 4 weeks postpartum in comparison to a single dose.	3/5 Americas (AMRO)
Bhaskaram [23]	vitamin A supplementation (200,000 IV)	102 women who did not receive any vitamin A supplements during pregnancy and have full term normal deliveries	a double blind controlled prospective study	To investigate the vitamin A status of breast fed infants, extent of cornea1 lesions and the impact of postnatal maternal vitamin A supplementation on their growth and vitamin A status.	Retinol	The mean values were significantly higher at 10 and 30 days in supplemented mothers compared to control group.	2/5 South East Asia (SEARO)
Canfield [24]	Mothers in Group I received 90 mg β-carotene as red palm oil concentrate. Mothers in Group II received capsules containing 90 mg purified β-carotene (BASF) and Group III mothers received placebo capsules identical in appearance to those containing β-carotene.	Ninety-eight mothers mean age: 26.0 ± 6.5	randomised clinical trial	To investigate the effect of β- carotene added to the diets of mothers as red palm oil or supplements on the vitamin A status of mothers and their nursing infants in a marginal barrio of Tegucigalpa, Honduras.	Carotenoids and retinol	Changes in milk concentrations of α-carotene (*P* < 0.01) and β-carotene (*P* < 0.02) before and after supplementation were significantly different between the three experimental groups. Increases in β- carotene concentrations were greater for the palm oil group (2.5 fold, *p* < 0.0001) than for the β-carotene supplement group (1.6 fold, *p* < 0.006) relative to placebo.	2/5 Americas (AMRO)
Canfield [25]	B-carotene beadlets (30-mg capsules)	Forty-four lactating mothers who had vitamin-A–poor diets. Mean age 23.7 ± 6.4 years.	randomised clinical trial	Investigation the effect of short-term b-carotene supplementation of lactating mothers on maternal milk.	Milk retinol and carotenoids	B-Carotene supplementation markedly elevated maternal serum and milk b-carotene concentrations (nine- and sevenfold, respectively). Maternal serum and milk retinol were unchanged in response to the treatment.	2/5 Americas (AMRO)
Darboe [26]	vitamin A as retinyl palmitate	197 mothers With a child weighing more than 2200 g and delivering over 37 weeks	Randomised, double blind, placebo controlled trial.	To compare the efficacy of the International Vitamin A Consultative Group early high-dose protocol with that of the WHO protocol by the assessment of adverse events at dosing, maternal and infant vitamin A concentrations, mucosal integrity, growth and morbidity patterns, and measurements of infant immunity.	vitamin A	At 1 month postpartum, there was a non-significant trend towards higher levels of breast milk retinol in the high dose group than in the WHO group.	4/5 Africa (AFRO)
Dijkhuizen [27]	All women received iron and folic acid (30mgiron as ferrous fumarate/d and 0.4 mg pteroylglutamic acid/d). In addition, one group of women received β –carotene (4.5 mg as water-soluble granulate/d; β-carotene group), one group received zinc (30 mg zinc as sulfate/d; zinc group), one group received β –carotene plus zinc (4.5 mg β –carotene and 30 mg zinc/d; β –carotene zinc group), and one group received only iron and folic acid (control group).	Pregnant women (*n =* 170). Mean age 25.1 ± 5.6 years.	A double-blind, placebo-controlled study	To investigates whether supplementing women during pregnancy with β-carotene and zinc, in addition to the standard supplementation with iron and folic acid, can improve vitamin A and zinc status of mothers and newborns 1 and 6 months postpartum.	Breast-milk b-carotene Retinol Zinc	Breast-milk β-carotene concentrations were higher in all women supplemented with β –carotene, but breast-milk retinol concentrations were higher only in women who received β –carotene + zinc. Zinc concentrations did not differ among groups in mothers and infants. Six months postpartum, plasma retinol concentrations were higher in the women who received zinc during pregnancy than in women who did not.	4/5 South East Asia (SEARO)
Garcia-Guerra [28]	100 to 150% of the recommended dietary vitamin A	249 mothers with 23.0 ± 5.2 years.	A randomized controlled trial	To assess the impact of daily supplementation with multiple micronutrients during pregnancy on zinc, vitamin A and folate status during pregnancy and 1 month postpartum, zinc and vitamin A in cord blood, and breast milk retinol concentration at one month postpartum.	Retinol	Breast milk retinol concentration at one month postpartum didn’t differ between groups.	4/5 Americas (AMRO)
Gossage [29]	All mothers were randomly assigned to 4 weeks of supplementation with either β carotene (30 mg/d; *n* = 11) or placebo (*n* = 10) beginning on day 4 postpartum (day 0 of the study).	Twenty-one pregnant women who had breast-fed at least one infant, did not smoke, had not taken prenatal supplements.	randomized controlled trial	To investigate the effects of β -carotene supplementation on human milk composition.	Milk concentrations of β -carotene, the other carotenoids, retinol, or α-tocopherol.	Β carotene supplementation did not significantly change the milk concentrations of β -carotene, the other carotenoids, retinol, or α tocopherol.	2/5 Americas (AMRO)
Grilo [30]	A mega dose of 200,000 IU of retinyl palmitate.	33 voluntary postpartum women aged 18 to 35 years	quasi-experimental study	To investigate the effect of vitamin A supplementation on the retinol concentration in colostrums under fasting and postprandial conditions	retinol	After supplementation, the values were 89.5 (32.9–264.2) g/dL and 102.7 (37.3–378.3) g/dL in fasting and postprandial conditions in breast milk, respectively (*p* < 0.05), representing an increase of 14.7%.	3/5 Americas (AMRO)
Grilo [31]	The supplemented group received 200,000 IU of retinyl palmitate after the first colostrum collection.	Healthy puerperal women were randomly distributed into a control group (*n* = 44) and a supplemented group (n = 44).	prospective, controlled, randomised and parallel-design trial	To assess the influence of maternal retinyl palmitate supplementation on the levels of retinol and a-tocopherol in the colostrum and mature milk of healthy lactating women.	Retinol a-tocopherol	The colostrum retinol levels of the supplemented group increased significantly 24 h after the intervention (*P* < 0.001). However, the retinol levels in the mature milk of both groups did not differ (*P* > 0.05). Moreover, after maternal supplementation with vitamin A, the colostrums a-tocopherol level decreased by 16.4%, which is a significant reduction (*P* < 0.05). However, vitamin A supplementation did not affect the a-tocopherol level of mature milk (P > 0.05).	3/5 Americas (AMRO)
Idindili [32]	60,000 g vitamin A palmitate	780 newborn infants and their mothers	Randomised, double blind, placebo controlled trial.	To compare the safety and efficacy of the previously tested low-dose regimen 25,000 IU vitamin A palmitate on breast milk.	Vitamin A	There were no significant difference in breast milk concentration of vitamin A in high and low dose group.	5/5 Africa (AFRO)
Johnson [33]	Participants were given either seven doses of a placebo (n = 4) or seven doses of naturally occurring BC (*n* = 8).	Twelve healthy lactating women (1–8 months postpartum, 18–40 years old).	randomized controlled trial	To determine effect of continuous oral doses of b-Caroten on breast Milk composition.	Carotenoids b-Caroten isomers	In the experimental group, the mean maternal milk concentration of all-trans b-carotene significantly increased to seven times the baseline level by the end of the supplementation period. The maternal milk concentration of 9-cis b-carotene significantly increased to three times the baseline level by the end of the supplementation period.	2/5 Americas (AMRO)
Klevor [34]	Women were randomly assigned to receive either the multiple-micronutrient supplement (MMN) providing 18 micronutrients, including 800 mg retinol equivalents of vitamin A, or the lipid-based nutrient supplement (LNS) with the same nutrients as the MMN group, plus 4 minerals and macronutrients, until 6 mo postpartum; a control group received iron and folic acid during pregnancy and a placebo (calcium tablet) during the first 6 months postpartum.	1320 women during pregnancy (≤20 wk. of gestation to delivery) and the first 6 mo postpartum.	A randomized, partially double-blind, controlled trial	Assessing effect of daily supplementation with approximately the recommended daily intake of vitamin A in LNS or a multiple-micronutrient supplement (MMN) during pregnancy and the first 6 mo postpartum on breast milk retinol concentration at 6 mo postpartum.	Breast milk vitamin A	There were no significant differences in any of these outcomes among intervention groups and results did not change after controlling for significant covariates. We found no significant effect of daily low-dose vitamin A provided in LNS or MMN during pregnancy and the first 6 mo postpartum on breast milk retinol concentration at 6 mo postpartum**.**	5/5 Africa (AFRO)
Lietz [35]	control group (*n* = 30), the sunflower oil group (*n* = 30), and the red palm oil group (*n* = 30)	Ninety rural, pregnant Tanzanian women from 3 randomly selected villages were recruited during their third trimester.	randomized controlled trial	To efficacy of red palm oil in increasing retinol and provitamin A status in pregnant and lactating women.	carotenoid and retinol	Supplementation with red palm oil, which is rich in provitamin A, increased α- and β- carotene concentrations significantly in both plasma and breast milk. The difference in change in breast-milk retinol concentration between the red palm oil group and the control group was significant.	2/5 Africa (AFRO)
Lietz [36]	Red Palm Oil	56 Pregnant women (in their 3rd trimester, aged 18–45 years).	quasi-experimental study	To investigate the effect of maternal red palm oil supplementation throughout the 3rd trimester of pregnancy and the first 3 mo postpartum on carotenoid pattern in both plasma and breast milk.	Xanthophyll and Hydrocarbon Carotenoid	Red palm oil supplementation increases the milk concentrations of provitamin A carotenes without decreasing the milk concentrations of xanthophylls.	2/5 Africa (AFRO)
Martins [37]	a single dose of 200,000 IU vitamin A (retinyl palmitate)	66 mother–infant pairs, 33 mothers in control and 33 mothers in intervention group.	A double blind, placebo-controlled randomized clinical assay	Assess the impact of maternal supplementation with a single dose of retinyl palmitate on the vitamin A status of mother, breast milk and infant.	Serum and milk retinol	Reduction in breast milk retinol was observed in the control group compared with the pre-supplementation levels (1.93 and 1.34 mmol/l, respectively; P ≤ 0.0001) and to the post-supplementation levels of the supplemented group (1.56 mmol/l; *P* = 0.0003). There was significant difference in the prevalence of VAD in breast milk after supplementation, 55.6% (15/27) in the control group and 16.1% (5/31) in the supplemented group (*P* = 0.002).	3/5 Americas (AMRO)
Muslimatun [38]	One group received (*n* 5 88) a weekly supplement of iron (120 mg Fe as FeSO4) and folic acid (500 mg) and another (*n* 5 82) the same amount of iron and folic acid plus vitamin A [4800 retinol equivalents (RE)].	170 women with age 17–35 years.	A randomized double-blind, community-based trial	To investigate whether retinol and iron variables in breast milk and in serum postpartum were enhanced more with weekly vitamin A and iron supplementation during pregnancy than with weekly iron supplementation alone.	Fat, iron and vitamin A	Compared with the weekly iron group, the weekly vitamin A and iron group had a greater (P < 0.05) concentration of retinol in transitional milk (as mmol/L) and in mature milk (as mmol/g fat). However, no positive effects were observed on iron status and iron concentration in breast milk.	3/5 South East Asia (SEARO)
Nagayama [39]	Chlorella tablets.	Twenty healthy pregnant women (age range, 24–39 years.	randomized controlled trial	Investigation the effect of maternal supplementation with Chlorella on the carotenoid concentrations of breast milk.	carotenoids	Among the carotenoids detected in breast milk, lutein, zeaxanthin and b-carotene concentrations in the Chlorella group were 2.6-fold (p = 0.001), 2.7-fold (*p* = 0.001) and 1.7-fold (*p* = 0.049) higher, respectively, than those in the control group.	2/5 WPRO (Western Pacific Regional Office)
Rice [40]	a single dose of 200,000 international units [60,000 retinol equivalents (RE)] vitamin A followed by daily placebos (*n =* 74), (2) daily doses of b-carotene [7.8 mg (1300 RE)] (*n =* 73) or (3) daily placebos (*n* = 73) until 9 mo postpartum	220 women in three treatment group. b-carotene Vs Placebo Vs Vitamin A	Randomized double blinded clinical trial	To investigate the the effects of maternal postpartum vitamin A or b-carotene supplementation on maternal and infant serum retinol concentrations and breast milk vitamin A concentrations.	Vitamin A	Compared to placebos, vitamin A supplementation resulted higher milk vitamin A concentrations at 3 mo, but these improvements were not sustained. Women receiving b-carotene supplements produced breast milk with increasingly higher vitamin A concentrations from 3 to 9 mo, but the concentration was significantly different from the placebo group only at 9 mo.	5/5 South East Asia (SEARO)
Roy [41]	209 mmol retinol.	50 pregnant women in their last trimester aged 16 ± 35 year.	Randomized clinical trial	To evaluate the effect of vitamin A supplementation 24 h after delivery on breast milk retinol concentration.	serum retinol, Breast milk retinol	Mean serum retinol levels increased in the supplemented mothers at 2.77 (2.3, 3.2) compared to 1.15 (0.9, 1.4) mmol/l in controls (*P* < 0.05) and remained at a significantly higher level of 1.59 (1.4, 1.8) mmul/l compared to 1.33 (1.8, 1.5) mmol/l in the control group (P < 0.001) up to a period of three months.	2/5 Eastern Mediterranean (EMRO)
Stoltzfus [42]	300,000 IU vitamin A as retinyl palmitate (n = 77) 2. Mother received placebo (n = 76).	153 mothers and their infants. Mother received supplement (*n* = 77) mother received placebo (*n* = 76).	Randomised, double blind, placebo controlled trial.	To measure the effects of supplementing mothers postpartum with vitamin A and effects on their milks.	breast milk retinol concentration	The milk retinol concentrations of the vitamin A group were higher than those of the placebo group (*P* < 0.05).	5/5 South East Asia (SEARO)
Tomiya [43]	Group 1 e received one 200,000 IU (retinol palmitate) capsule 40 mg of vitamin E orally immediately after delivery and, 10 days after delivery, the second 200,000 IU (retinol palmitate) capsule 40 mg of vitamin E were given. Group 2 e received one 200,000 IU (retinol palmitate) capsule 40 mg of vitamin E orally immediately after delivery and, 10 days after delivery, the second “placebo” capsule containing 40 mg of vitamin E diluted in soybean oil were given.	158 women from 13 to 42 years of age.	A randomized, controlled, triple blind and hospital based clinical trial.	To determine if the 400,000 IU supplementation with retinol palmitate, immediately after delivery, promotes an additional effect in the concentrations of retinol in the human milk, when compared to the 200,000 IU supplementation.	Retinol	There was no significant difference between retinol concentrations in breast milk between treatment groups (400,000 IU vs 200,000 IU) in the studied period: 2 months (*p* = 0.790) and 4 months (*p* = 0.279).	5/5 Americas (AMRO)

**Table 2:** Summary effect of nutritional vitamins supplements on status of vitamin B human milk composition

First author surname, citation number	Type of Supplement	Characteristics of Participants	Type of study	Aim	Type of Nutrients Evaluated in Milk	Main findings	Jadad scale points and WHO divisions
Bates [44]	60 mothers living in two Gambian villages were given either 2 mg riboflavin or a placebo daily on a double-blind basis for 12 wk. Their riboflavin intake from dietary sources was about 0.5 mg/day	60 lactating mothers (mean age was 28.0 yr; their mean parity, 4. 1,)	Randomized double-blinded clinical trial	To evaluate the effect of a moderate increase in riboflavin intake on breast milk riboflavin level.	Riboflavin	Clinical signs associated with riboflavin deficiency improved more rapidly in the supplemented group; their breast milk riboflavin levels increased, and their infants’ activation coefficients (AC) were reduced, compared with those of the placebo group. After withdrawal of the supplement, the maternal and infants’ AC’s rose toward those of the placebo group.	4/5 Africa (AFRO)
Chang [45]	Mothers received pyridoxine (PN) supplements of 2.5, 4.0, 7.5, or 10.0 mg/d, respectively	Forty-seven infants born to healthy women were divided into four groups.	Randomized clinical trial	To assess maternal vitamin B6 intake and breast milk concentration of vitamin B6.	Vitamin B6	Mean vitamin B6 concentrations in breast milk were significantly lower for women supplemented with 2.5 mg PN.ehcl/d than for those supplemented with 4.0, 7.5, or 10.0 mg/d.	1/5 Americas (AMRO)
Duggan [46]	Daily oral dose of vitamin B-12 (50 mg) or a placebo identical in appearance.	One hundred eighty-three women were randomly assigned to receive vitamin B-12 and 183 to receive placebo.	A randomized, double-blind, placebo-controlled trial	To evaluate the effect of maternal supplementation of vitamin B-12 during pregnancy and lactation on maternal and infant biomarkers of vitamin B-12 status.	Milk vitamin B-12	At 6 wk. postpartum, median breast milk vitamin B-12 concentration was increased in vitamin B-12–supplemented women than placebo group (*P* < 0.0005). Oral supplementation of urban Indian women with vitamin B-12 throughout pregnancy and early lactation significantly increases vitamin B-12 status of mothers and infants.	5/5 South East Asia (SEARO)
Hampel [47]	Lipid-based nutrient supplements (LNS)	(*n* = 258) or 6 (*n* = 104), and 24 weeks (*n* = 362) from HIV infected Malawian mothers.	Randomized clinical trial	To investigate the contribution of each thiamin and riboflavin vitamer and the effect of lipid-based nutrient supplements (LNS) on the vitamer distribution at early and later stages of lactation.	Thiamin vitamers, Riboflavin and FAD, Thiamin-pyrophosphate (TPP),	Lipid-based nutrient significantly increased Thiamin-monophosphate and free thiamin only at 2 weeks compared to the control. Free riboflavin was consistently and significantly increased with LNS versus control.	1/5 Africa (AFRO)
Hampel [48]	Thiamin, riboflavin, niacin, and vitamins B-6, B-12, A, and E.	18 healthy women (aged 18–26 years)	Randomized clinical trial	To evaluate the effects of sample collection protocols, variations in circadian rhythms, subject variability, and acute maternal micronutrient supplementation on milk vitamin concentrations.	Thiamin, riboflavin, niacin, and vitamins B-6, B-12, A, and E and fat were measured in each sample	No significant differences were observed for thiamin and vitamins B-12, A, E, and A. Vitamin B-6 concentrations increased linearly after supplements were consumed, whereas milk concentrations of riboflavin increased on day 3 and 4 compared with day 1.	1/5 South East Asia (SEARO)
Siddiqua [49]	250 μg/day B12 or a placebo throughout pregnancy and 3-month postpartum along with 60 mg iron + 400 μg folate.	68 women age 18–35 years.	Randomized clinical trial	To assess the effect of B12 supplementation in pregnancy and lactation on alleviation of anemia, and improvement of B12 status and vaccine-specific immunity in mothers and infants.	Vitamin B12	Supplementation increased B12 in plasma, colostrums and breast milk (*p* < 0.05) and lowered methylmalonic acid in neonates, mothers and infants at 3 months (*p* < 0.05). Supplementation with 250 mg/day B12 during pregnancy and lactation substantially improved maternal, infant and breast milk B12 status.	4/5 South East Asia (SEARO)
Styslinger [50]	Pyridoxine HCI	Twenty-four healthy, lactating women with full-term, healthy infants and their ages ranged from 20 to 36 years.	Randomised-controlled trial	To examine the effects of such changes on the breastfed infants’ intake of vitamin B-6.	Vitamin b6	A significant (*p* < 0.001) positive correlation (r = 0.80) was found between maternal intake and the level of the vitamin in milk. The mean vitamin 8–6 content in milk of subjects supplemented with 20.0 mg vitamin 8–6/day was significantly higher (p < 0.05) than that for any other group.	1/5 Americas (AMRO)
Thomas [51]	Vitamin A, Vitamin D, Vitamin E, Vitamin C, Folic acid, Thiamin, Riboflavin, Niacin, Vitamin B6, Vitamin B12, Calcium, Iodine, Iron, Magnesium.	17 mothers at the end of gestation (18 to 35 years of age)	Randomised-controlled trial	To study the effects of vitamin supplements and/or diet on the levels of vitamin C, vitamin B6, and vitamin B12 in milk and blood of lactating women.	Vitamin B6, B12 and C	The vitamin B6 level in breast milk of the unsupplemented group of mothers was significantly lowers (P < 0.05) than the supplemented group of women at 5 to 7 days postpartum. Vitamin B12 concentration in milk of nonsupplemented mothers at 43 to 45 days postpartum was significantly lowers (P < 0.05) than the supplemented group of women at 43 to 45 days postpartum.	1/5 Americas (AMRO)

**Table 3:** Summary effect of nutritional vitamins supplements on status of vitamin C human milk composition

First author surname, citation number	Type of Supplement	Characteristics of Participants	Type of study	Aim	Type of Nutrients Evaluated in Milk	Main findings	Jadad scale points and WHO divisions
Byerley [52]	25 lactating women administered 90 mg of ascorbic acid for 1 day followed by 250, 500 or 1000 mg/day for 2 days or unsupplemented for 1 day followed by either 0 or 90 mg ascorbic acid supplement for 2 days	25 well-nourished lactating women from 20 to 36 years old.	Randomized clinical trial	To assess effect of maternal intake of vitamin C on the vitamin C concentration in human milk and on the vitamin C intakes of breast-fed infants.	Vitamin C of human milk	Total maternal intakes of vitamin C, which exceeded 1000 mg/day or 10-fold the RDA for lactation (100 mg/day), did not significantly influence the vitamin C content in milk or the vitamin C intakes of infants.	1/5 Americas (AMRO)
Daneel-Otterbech [53]	Effervescent tablets (1000 mg ascorbic acid)	Apparently healthy, lactating women	Randomised-controlled trial	To compare human milk ascorbic acid content in European and African women and to evaluate the influence of increased ascorbic acid intake on human milk ascorbic acid output.	Ascorbic acid	Ascorbic acid (AA) supplementation (1000 mg/d for 10 d) increased mean human milk AA from 19 to 60 mg/kg (*P* ≤ 0.001) and from 60 to 70 mg/kg (*P* ≤ 0.03) in 18 African and 10 European women, respectively. In 11 African women, mean human milk AA increased from 17 to 36 mg/kg (*P* ≤ 0.001) after intake of 100 mg AA/d for 10 d.	1/5 Europe (EURO)

**Table 4:** Summary effect of nutritional vitamins supplements on status of vitamin D human milk composition

First author surname, citation number	Type of Supplement	Characteristics of Participants	Type of study	Aim	Type of Nutrients Evaluated in Milk	Main findings	Jadad scale points and WHO divisions
Ala-Houhala [54]	Half of the mothers were supplemented daily throughout the study with one tablet containing 1000 IU (25 mg) of ergocalciferol. Throughout	20 mothers who delivered babies in the winter and from 20 mothers who delivered in the summer.	Randomized clinical trial	To investigate the possible effects of supplementing breast-feeding mothers with vitamin D and of the seasons on the concentrations of antirachitic sterols, ie, 25-hydroxyvitamin D (25- [OH]D) and vitamin D, in milk.	25 (OH) vitamin D	Supplementation had no effect on vitamin D levels. Milk 25-(OH) D levels of mothers receiving either 1000 or 2000 IU (25 or 50 g) vitamin D/d was significantly higher than those of un supplemented mothers in February and April.	1/5 Europe (EURO)
Basile [55]	Mothers were randomized to receive either 2000 or 4000 IU vitamin D supplementation per day. Groups 1 and 2 received 1600 and 3600 IU per day vitamin D2, respectively, in an oral suspension. Both groups received additional multivitamin capsules containing 400 IU vitamin D3 and were instructed to take them daily.	25 Lactating mothers (mean age: 30.6 ± 4.6)	Prospective, double-blinded, randomized Controlled trial	To investigate breast milk [Ca] as a function of vitamin D supplementation regimen.	Circulating and Milk concentrations of vitamin D2, vitamin D3, 25(OH)D2, and 25(OH)D3	Mean maternal and infant total circulating 25(OH) D levels had statistically significant increases during the 3 months of high-dose vitamin D supplementation. Mothers in group 1 (who received 1600 IU/day vitamin D2 and 400 IU/day vitamin D3) exhibited increases in total circulating concentrations of 25(OH) D from baseline to 3 months (*p* ≤ 0.002).	4/5 Americas (AMRO)
Ketha [56]	Vitamin D3 as a single oral dose of 150,000 IU (*N* = 20), or 5000 IU daily (N = 20) for 28 days.	40 lactating females, ages 24–40 years, with a singleton infant between the ages of 1 and 6 months.	Randomized controlled trial	To investigate the effect of bolus versus daily vitamin D3 dosing regimens on temporal changes in 25(OH)D3/24,25(OH)2D3 ratio in a group of breast feeding mothers	Serum vitamin D3, 25(OH) D3, 24,25(OH)2D3, 1,25(OH)2D3, and breast milk vitamin D3.	Greater production of 24, 25(OH) 2D3 resulting from a single, high dose bolus of vitamin D than with a daily dose of vitamin D over the course of 28 days.	2/5 Americas (AMRO)
Niramitmahapanya [57]	One group received vitamin D3 1800 IU/d supplementation for 6 weeks, and members of the other group were given a placebo.	200 mothers at the third trimester (delivered singleton infants at term > 37 weeks).	Randomized double blinded control trial	To investigate whether vitamin D3 supplementation (1800 IU/day) can improve breastfed serum of infants and breast milk 25 (OH) D levels.	25 (oh) d	At 6 weeks, maternal 25 (OH) D levels had increased significantly in the vitamin D group (VD) 68.30 + 15.40 nmol/L compared to 55.15 + 13.57 nmol/L in the placebo group (*p* < 0.001).	4/5 South East Asia (SEARO)
Oberhelman [58]	Participants received oral cholecalciferol (vitamin D3) 5000 IU/d for 28 days or 150,000 IU once.	Forty mothers, mean age: 30.3 ± 2.9	Randomized clinical trial	To determine if a single monthly supplement was as effective as a daily maternal supplement in increasing breast milk vitamin D to achieve vitamin D sufficiency in their infants.	Cholecalciferol	In mothers given daily cholecalciferol, concentrations of serum and breast milk cholecalciferol attained steady levels of 18 and 8 ng/ ml, respectively from day 3 through 28. In mothers given the single dose, serum and breast milk cholecalciferol peaked at 160 and 40 ng/ml, respectively at day 1, before rapidly declining.	2/5 Americas (AMRO)
Wall [59]	A placebo group, a group who received one dosage of daily oral vitamin D3 (1000 IU), or a group who received 2 dosages of daily oral vitamin D3 (2000 IU)	48 pregnant mothers and their infants	A randomized, double blinded, Placebo-controlled trial	To examine the effect of vitamin D supplementation during pregnancy on breast-milk VDA in the first 2 mo of lactation.	Concentration Of vitamin d2, vitamin d3, 25(oh)d2, and 25(oh)d3 in Breast milk	Maternal vitamin D supplementation during pregnancy of 2000 IU/d (compared with 1000 IU/d and with a placebo) results in a higher VDA of breast milk ≥2 mo postpartum.	3/5 WPRO (Western Pacific Regional Office)

**Table 5:** Summary effect of nutritional vitamins supplements on status of vitamin E human milk composition

First author surname, citation number	Type of Supplement	Characteristics of Participants	Type of study	Aim	Type of Nutrients Evaluated in Milk	Main findings	Jadad scale points and WHO divisions
Clemente [60]	A control group without treatment, a group receiving an acetate capsule with natural RRR-a-TOH (GNAT), and a group receiving an acetate capsule with synthetic all-rac-a-TOH (GSINT)	109 healthy lactating women ages 18 to 40 year.	A randomized double-blind clinical trial	Evaluate levels of vitamin E in human colostrums when lactating mothers were given supplements of either natural or synthetic forms of alpha-tocopherol.	A-tocopherol	Women who received supplementation had higher concentrations of a-TOH in colostrums than the control group, with 57 and 39% increases in women supplemented with the natural and synthetic forms of a-TOH, respectively.	4/5 Americas (AMRO)
Gaur [61]	The 3 groups were as follows: 1) 45.5 mg all-rac-a-TAc (ARAC), 2) 22.8 mg all-rac-a-Tac + 20.1 mg RRR-a-tocopherol (MIX), and 3) 40.2 mg RRR-a-tocopherol (RRR	Eighty-nine mothers aged 19–40 years.	Prospective, randomized, double-blinded, and placebo-controlled	To elucidate the effect of a-tocopherol supplementation with RRR-a-tocopherol, all-rac-a-tocopherol acetate (Tac), or a mixture (combination of RRR-a-tocopherol and all-rac-a-Tac) on the a-tocopherol stereoisomer distribution in plasma and milk.	A-tocopherol structural isomers and a-tocopherol stereoisomers	There were no significant treatment group or time-dependent changes in milk or plasma a, g, or d-tocoph erol. Supplementation changed both milk and plasma percentage RRR-a-tocopherol (*P* < 0.05) and percentage non-RRRa-tocopherol (P < 0.05). In the RRR group, percentage RRR-a-tocopherol increased in milk (mean 6SEM: 78 62.3% compared with 82 61.7%) (P < 0.05)	3/5 Americas (AMRO)
Johnson [33]	Beta carotene	Healthy lactating women (1–8 mo postpartum, 18–40 y old)	Randomized clinical trial	Examination of the concentration of BC isomers in breast milk and buccal mucosa cells after continuous oral doses of BC isomers is a simple, non-invasive method.	Beta carotene	The changes in breast milk concentration of all-trans BC in response to a continuous oral dose of BC followed a pattern similar to that for serum. A significant increase in concentration was observed by d 3 (*P* < 0.009) and steadily increased to six times the baseline level (d 1) by the end of the supplementation period (d 8, *P* < 0.0001).	2/5 Americas (AMRO)
Kanno [62]	d-α.Tocopherol	Mother, who had delivered a low-birth-weight infant (1975 g) after 38 weeks of gestation	Quasi experimental study	To investigated the transfer of a-T into the breast milk of a lactating mother who was orally administered with α.Tocopherol	α.Tocopherol	By a single dose of α. Tocopherol, the content of α. Tocopherol per lipid was increased by a maximum of 7-fold and the ratio of α. Tocopherol equivalent/PUFA was markedly improved, although the total amount of α. Tocopherol transferred into the milk was small.	1/5 WPRO (Western Pacific Regional Office)
Medeiros [63]	A single oral dose of 400 IU natural vitamin E.	Participants were women aged between 18 and 45 years.	Randomized clinical trial	To assess the effect of vitamin E supplementation on the α-tocopherol concentrations of colostrums, transitional milk and mature milk of women who had given birth prematurely.	Α-Tocopherol	Breast milk α-tocopherol concentrations increased by 60% 24 h after supplementation in the intervention group and did not increase at all in the control group. Α-Tocopherol concentration of the transitional milk in the supplemented group was 35% higher compared with the control group.	2/5 Americas (AMRO)
Melo [64]	capsules containing 400 international units (IU) of alpha-tocopherol	99 healthy pregnant women; mean age was 24 ± 6 years.	Randomized clinical trial	To evaluate the effect of maternal supplementation with vitamin E on the concentration of α tocopherol in colostrums and its supply to the newborn.	a-tocopherol	After supplementation, the mean concentration of -tocopherol in the mothers’ colostrums was 1650.6 ± 968.7 and 2346.9 ± 1203.2 mg/dL in the CG and SG groups, respectively. However, the mean αtocopherol concentrations in supplemented women increased the concentration of αtocopherol secreted in colostrums by 61% (*p* < 0.001).	3/5 Americas (AMRO)

**Table 6:** Summary effect of nutritional vitamins supplements on status of vitamin K human milk composition

First author surname, citation number	Type of Supplement	Characteristics of Participantss	Type of study	Aim	Type of Nutrients Evaluated in Milk	Main findings	Jadad scale points and WHO divisions
Bolisetty [63]	2.5 mg phylloquinone (vitamin K1) orally daily for 2 weeks	Six healthy lactating mothers who gave birth to preterm infants at a median post conceptional age of 29.5 (range 26–30) weeks.	Randomized clinical trial	To raise the vitamin K content in the breast milk to levels recommended for infant formulae by RDA and to look at day-to-day variation in the breast milk vitamin K levels with maternal supplementation of vitamin K.	phylloquinone vitamin K1	Phylloquinone levels in the breastmilk increased from a baseline of 3 ± 2.3 ng ml^− 1^ to 22.6 ± 16.3 ng ml^− 1^ (mean ± SD) after the first dose (*p* < 0.05); a gradual increase was noted until phylloquinone levels reached a plateau of 64.2 ± 31.4 ng ml^− 1^ after the sixth daily dose.	2/5 WPRO (Western Pacific Regional Office)
Greer [65]	Ten mothers received 2.5 mg/d oral phylloquinone, and 10 mothers received 5.0 mg/d oral phylloquinone.	Twenty exclusive breastfeeding mothers.	longitudinal, randomized, double-blind, placebo-controlled	To increase the phylloquinone (Vitamin K1) concentration of human milk with maternal oral phylloquinone supplements.	Phylloquinone	Both 2.5 and 5.0 mg/d phylloquinone significantly increased the phylloquinone content of human milk at both 2 and 6 weeks. As expected, 5.0 mg had a greater effect.	3/5 Americas (AMRO)
Kries [66]	Vitamin K1	Nine mothers (age of the mothers ranged between 17 and 34 yr, mean 24 yr)	Randomized clinical trial	To assess the effect of Vitamin KI Supplements on maternal milk.	Vitamin K1	To test the influence of diet, mothers were given oral supplements of vitamin K I. Doses of 0.5–3 mg produced substantial rises in breast milk vitamin K I with peak levels between 12 and 24 h. In one mother in whom the milk sampling was standardized, a dose-response relationship was observed.	1/5 Europe (EURO)

**Table 7:** Summary effect of nutritional vitamins supplements on status of multiple vitamins human milk composition

First author surname, citation number	Type of Supplement	Characteristics of Participants	Type of study	Aim	Type of Nutrients Evaluated in Milk	Main findings	Jadad scale points and WHO divisions
Canfield [67]	Purified b-carotene in capsules	Five healthy mothers, between the ages of 23 and 36 years.	Randomized clinical trial	Investigate changes in concentrations of milk and serum carotenoids, retinol, and a-tocopherol of five healthy women over a 28-d supplementation trial with 30 mg b-carotene and for 4 wk. thereafter.	milk a-tocopherol and retinol	B Carotene supplementation increased mean b-carotene concentrations in milk and serum 6.4- and 7.4-fold, respectively. Concentrations of other major carotenoids, retinol, and a-tocopherol did not change substantially in either milk or serum.	3/5 Americas (AMRO)
Garcia [68]	One retinyl palmitate capsule (200,000 UI)	73 Healthy parturient women, control (*n* = 37) and supplemented (*n* = 36).	Randomized clinical trial	To determine the effect of maternal supplementation with a megadose of retinyl palmitate in the immediate post-partum on a-tocopherol concentration in the colostrums.	Retinol, a-tocopherol	A significant increase (*P* = 0.00) was observed in colostrums retinol in the supplemented group 24 h after administration of the retinyl palmitate capsule. A significant increase (*P* = 0.04) was also found in colostrums a-tocopherol concentration after vitamin A supplementation.	1/5 Americas (AMRO)
Gossage [29]	B-carotene (30 mg/d; *n* = 11) or placebo (*n* = 10) beginning on day 4 postpartum (day 0 of the study).	Twenty-one pregnant women were recruited during their last trimester.	Randomized clinical trial	To assess milk carotenoid concentrations during days 4–32 postpartum and the effects of maternal B-carotene supplementation.	lutein + zeaxanthin, b-cryptoxanthin, lycopene, a-carotene, B-carotene, retinol, and a-tocopherol.	B-carotene supplementation did not significantly change the milk concentrations of B –carotene, the other carotenoids, retinol, or tocopherol. There were no significant overall effects of carotene supplementation on milk concentrations of lutein, cryptoxanthin, lycopene, or carotene.	2/5 Americas (AMRO)
Sherry [69]	Either 0 mg/d of lutein (placebo), 6 mg/d of lutein (low-dose), or 12 mg/d of lutein (high-dose).	Eighty-nine lactating women 4–6 wk. postpartum.	A multisite, prospective, randomized, placebo controlled dose-response study	To determine the impact of lutein supplementation in the breast milk and plasma of lactating women and in the plasma of breast-fed infants 2–3 mo postpartum.	Carotenoids, Lutein, zeaxanthin	Total lutein + zeaxanthin concentrations were greater in the lowand high-dose–supplemented groups than in the placebo group in breast milk (140 and 250%, respectively; *P* < 0.0001)	5/5 Americas (AMRO)
Webb [70]	(1) vitamin A and b-carotene (VABC: 5000 IU (1500 mg retinol activity equivalents) of preformed vitamin A plus 30 mg of b-carotene), (2) multivitamins (MV) that did not include vitamin A and b-carotene (20 mg of thiamine, 20 mg of riboflavin, 25 mg of vitamin B6, 100 mg of niacin, 50 mg of vitamin B12, 500 mg of vitamin C (purified L-ascorbic acid), 30 mg of vitamin E RRR-a- tocopherol acetate) and 0.8 mg of folic acid), (3) MV that included vitamin A and b-carotene (same doses as above), or (4) placebo.	1078 HIV-infected pregnant women.	A randomized, double-blind, placebo-controlled trial	To assess the impact of daily vitamin supplementation during pregnancy and lactation on concentrations of retinol, b-carotene, a-carotene, a-tocopherol, g-tocopherol and d-tocopherol in breast milk.	retinol, total b-carotene, a-carotene, a-tocopherol, d-tocopherol and g-tocopherol	Women who received VABC had significantly higher concentrations of breast milk retinol, b-carotene and acarotene at all-time points during the first year postpartum compared to women who did not receive VABC. Supplementation with VABC did not influence concentrations of a-, g- or d-tocopherol from delivery to 1 year postpartum.	4/5 Africa (AFRO)

**Table 8:** Summary effect of nutritional minerals supplements on status of Zinc human milk composition

First author surname, citation number	Type of Supplement	Characteristics of Participants	Type of study	Aim	Type of Nutrients Evaluated in Milk	Main Findings	Jadad scale points and WHO divisions
Chierici [71]	20 mg zinc sulfate, 2 mg copper sulfate and 116 mg potassium iodide.	32 non-smokers, non-vegetarian, with normal weight gain during pregnancy mothers.	Randomized controlled trial	To determine the effect of dietary zinc, copper and iodine supplements on the milk concentration.	Mineral content of zinc, copper and iodine	The milk zinc concentration declined significantly over the study period for all lactating subjects. There was no significant difference in the rate of decline between the women who started supplementation during lactation and those who were not supplemented.	1/5 Europe (EURO)
Krebs [15]	15 mg of zinc (as ZnSO_4_7H_2_O).	39 women who did not receive a zinc supplement and 14 women who received daily zinc supplement.	Randomized controlled trial	To calculate dietary zinc intakes, evaluate maternal zinc nutritional status, and determine zinc concentrations in milk.	Zinc	The rate of decline in milk zinc during lactation was significantly less for the supplemented group compared to that of the control group (*p* = 0.02). It is concluded that milk zinc concentrations are influenced by maternal zinc intake.	2/5 Americas (AMRO)
Shaaban [72]	10 mg/d of Zn sulfate capsules.	60 primiparous lactating mothers.	Randomized controlled trial	To determine effect of maternal Zn supplementation on maternal and infant Zn stores.	Zinc	Zn supplementation caused significantly higher maternal hair, nail, and breast milk Zn levels. In conclusion, Zn supplementation for lactating women positively influenced breast milk Zn concentrations and maternal body stores although it had no significant influence on the infants’ physical growth.	2/5 Eastern Mediterranean (EMRO)

**Table 9:** Summary effect of nutritional minerals supplements on status of selenium human milk composition

First author surname, citation number	Type of Supplement	Characteristics of Participants	Type of study	Aim	Type of Nutrients Evaluated in Milk	Main Findings	Jadad scale points and WHO divisions
Dodge [73]	50 mg selenium daily as selenomethionine or a placebo.	Twenty-two healthy young women between the ages of 20–30 years.	Single blind clinical trial	To evaluate the effect of selenium supplementation on the concentration of breast milk fatty acids in lactating women.	selenium and activity of glutathione peroxidase (Gpx)	Selenium concentration of breast milk was significantly increased by the supplementation (*P* = 0.0001 and 0.003, respectively), but glutathione peroxidase activity was unchanged. The selenium supplement also significantly increased the concentration of polyunsaturated fatty acids in breast milk (P = 0.02), especially linoleic acid (*P* = 0.02), and decreased the concentration of saturated fatty acids (P = 0.04).	3/5 WPRO (Western Pacific Regional Office)
Dylewski [74]	sodium selenate at 20 lg/day	23 lactating women who had not smoking, having no pregnancy complications, having an infant born at term (37 to 40 weeks).	Randomized clinical trial	To evaluate the impact of supplemental selenium as sodium selenate at 20 lg/day on maternal milk.	Selenium	Selenium supplementation increased milk Se from 3 (295 ± 18 nmol/L; 23 ± 7 ng/ mL) to 6 months (417 ± 39 nmol/L; 32 ± 14 ng/mL) postpartum (P ≤ 0.01)	1/5 Americas (AMRO)
Flax [75]	lipid-based nutrient supplements (LNS) that contained 1.3 times the Recommended Dietary Allowance of sodium selenite, antiretroviral drugs (ARV), LNS and ARV, or a control.	526 HIV-infected Malawian mothers and their uninfected infants attended in the Breastfeeding, Antiretrovirals, and Nutrition study	Randomized clinical trial	To determine the effects of lipid-based nutrient supplements maternal plasma and breast-milk selenium concentrations.	Selenium concentrations	Selenite supplementation of women was not associated with a change in their plasma or breast-milk selenium concentrations.	3/5 Africa (AFRO)
Moore [76]	Two groups and given either a placebo (n = 10) as yeast or selenium-enriched yeast tablets (n = 11)	21 pregnant women between the ages of 20 and 30 years living in Xichang County, China, a rural area in this country with historically low selenium intake	a single-blind, placebo-controlled, intervention study	To evaluate the effect of selenium supplementation on plasma and milk selenium concentrations and GPX activity over time after parturition.	Selenium	The milk selenium levels were higher in supplemented women but there were no differences in the milk GPX activity between the two groups of women. The plasma a-tocopherol concentrations declined after parturition in both groups but no differences were found between the two groups of women.	3/5 WPRO (Western Pacific Regional Office)
Trafikowska [77]	200 mg Se/day in the form of yeast-Se and sodium selenate.	Sixty seven lactating healthy women with aged 19 to 39 years (mean 26.7 years).	Randomized clinical trial	To determine the effect of selenium supplementation to lactating women on milk Se concentrations.	Selenium glutathione 1eroxidise (GSH-Px)	After 1 month in both groups SE level reached a plateau at 14–16 mglL. In both Se-supplemented groups the levels increased significantly reaching a plateau of 14–16 mg/L after 1 month. The difference was significantly higher than controls in the yeast-Se group (P < 0.0001) and the selenite-Se supplemented group (*P* < 0.01).	1/5 Europe (EURO)
Trafikowska [78]	Sixteen lactating women were supplemented for 3 months with 200 pg Seday-‘in the form of SeY. The supply was started 3–4 weeks postpartum.	16 healthy lactating mothers.	Randomized clinical trial	To assess the ability of maternal supplementation with Se-enriched yeast (SeY) to influence the Se status of lactating women and infants, mother’s milk, and additionally to estimate the infant’s dietary Se intake.	Se and GSH-Px activities of red cell haemolysate.	Supplementation of lactating mothers with selenium-enriched yeast increases rapidly and significantly the Se concentration and glutathione 2eroxidise activity in maternal blood components. Se concentration in milk is also significantly elevated. After 1 month the mean Se intakes by breastfed infants were greater than the recommended dietary allowance of 10 pg day-‘for infants from birth to ***6*** months of age.	1/5 Europe (EURO)

**Table 10:** Summary effect of nutritional minerals supplements on status of Iron human milk composition

First author surname, citation number	Type of Supplement	Characteristics of Participants	Type of study	Aim	Type of Nutrients Evaluated in Milk	Main Findings	Jadad scale points and WHO divisions
Breymann [79]	A single dose of 100 mg intravenous iron sucrose.	Ten healthy lactating mothers with functional iron deficiency 2–3 days after delivery.	Randomized controlled trial	To study the transfer of parenteral iron sucrose into maternal milk in the postpartum period.	Milk iron	No significant difference between the groups was found on any study day as well as in the mean change from baseline over all four days. We could not show transfer of iron-sucorose into maternal milk for the given dosage.	1/5 Europe (EURO)
Holm [80]	Single dose of intravenous 1200 mg iron isomaltoside or oral iron at a mean daily dose of 70.5 mg.	65 women with sufficient breast milk.	Randomized controlled trial	To compare the iron concentration in breast milk after a single high dose of intravenous iron isomaltoside or daily oral iron for postpartum haemorrhage.	Total iron concentration in breast milk	The mean (±SD) iron concentration in breast milk in the intravenous and oral groups was 0.72 ± 0.27 and 0.40 ± 0.18 mg/L at three days (*p* < 0.001) and 0.47 ± 0.17 and 0.44 ± 0.25 mg/L after one week (*p* = 0.64).	3/5 Europe (EURO)
Yalcın [81]	80 mg of elementary iron as ferrous sulfate.	47 Healthy mothers were enrolled in the study 10 to 20 days after delivery.	A prospective, placebo controlled, double-blinded, and randomized intervention	To determine the factors that affect milk iron content at the second week of lactation and whether supplementation to lactating mother with iron might increase breast milk iron content between 2 weeks and 4 months postpartum.	Iron and zinc	Iron supplementation to lactating nonanemic mothers did not change milk iron content and the decline in milk iron content and milk-to-serum iron ratio. Milk iron content and milk-to-serum iron ratio of iron could be regulated by active transport in cooperation with maternal iron status.	2/5 Europe (EURO)
zapata [73]	Iron sulfate (FeSO4.7H20) tablets containing 40 mg of Fe each.	Twenty-eight volunteer nursing women Their ages ranged from 19 to 35 years (average of 27);	Randomized controlled trial	To evaluate longitudinally the effect of moderate maternal iron supplementation during the first 3 months of lactation on milk iron levels and iron related milk components.	Concentrations of iron and zinc, lactoferrin, total iron-ligands in milk were measured	Iron supplementation did not alter significantly iron and zinc levels in milk and the low iron to lactoferrin ratio was maintained, thus preserving the important functions of lactoferrin for the infant organism. However, iron supplementation increased total iron ligands in milk as measured by the total iron-binding capacity and increased the proportion of lactoferrin in total protein secreted. Also, lactoferrin levels tended (*P* = 0.059) to be higher in milk of the supplemented women.	2/5 Americas (AMRO)

### Risk of bias assessment

We assessed the quality of the included studies using the risk of bias assessment tools developed by the Cochrane Collaboration [1], covering the following six domains: sequence generation, allocation concealment, blinding, incomplete data, selective reporting, and other bias. Two reviewers independently conducted the risk of bias evaluation and resolved any disagreement by discussion with a third reviewer. The reviewers’ judgment is categorized as ‘Low risk’, ‘High risk’ or ‘Unclear risk’ of bias.

RevMan (version 5.3) software was used for graphical display of risk of bias of included studies.

### Data extraction and abstraction

**Fig. 3:**
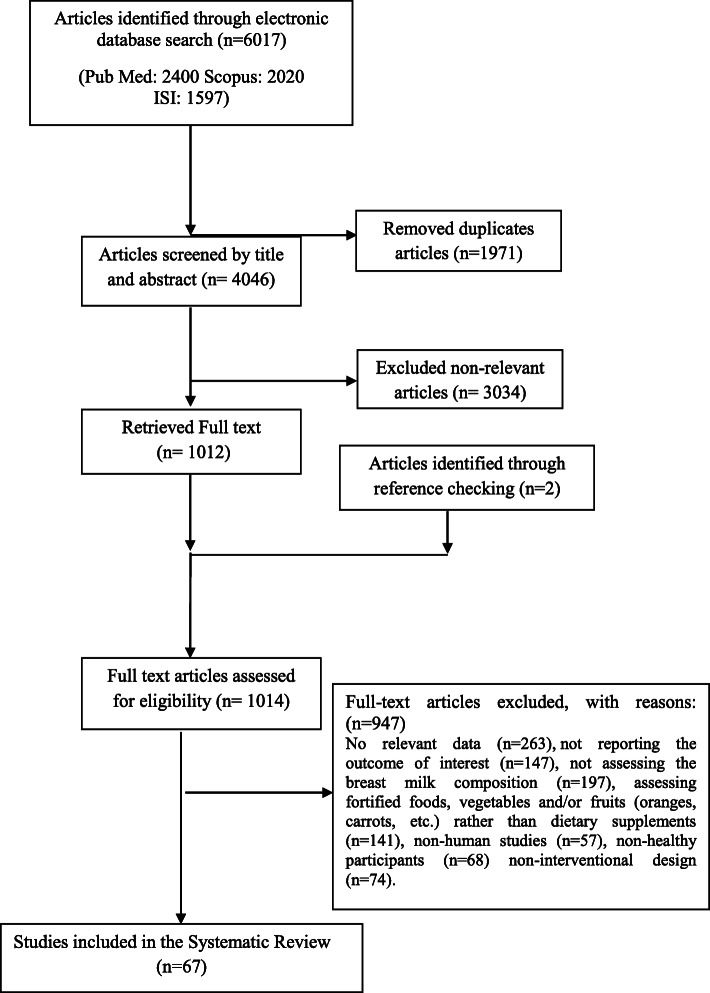
Papers search and review flowchart for selection of primary studies

We retrieved 4046 unique references after removing duplicates (in the basic search 1971 articles were duplicates that were found and removed using EndNote, Fig. 3). Of them, 3034 were excluded on the basis of the title and abstract. For the remaining 1012 articles, the full text was retrieved and critically reviewed. After the selection process, 67 papers were included in this systematic review.

Two independent reviewers (MK and RS) screened the titles and abstracts of papers, which were identified by the literature search, for their potential relevance or assessed the full text for inclusion in the review. In the case of disagreement, the discrepancy was resolved in consultation with an expert investigator (RK).

Two reviewers abstracted the data independently (MK and RS). The required information that was extracted from all eligible papers was as follows: data on first author’s family name, year of publication, country of the study, type of supplement or food, characteristics of participants, type of study, aim, type of nutrients evaluated in the milk and the main findings of studies. Accordingly, because of the importance of the study areas, as shown in Tables 1, 2, 3, 4, 5, 6, 7, 8, 9 and 10, we have provided the WHO regions for countries where the studies were conducted. These areas including; Western Pacific Regional Office (WPRO), South East Asia (SEARO), Europe (EURO), Eastern Mediterranean (EMRO), Americas (AMRO), Africa (AFRO).

## Results

In total, 67 paper were included in the current review; zinc (3 papers) [15, 71, 72], iron (4 papers) [79–82], selenium (6 papers) [73–78], vitamin A (26 papers) [18–43], vitamin B (8 papers) [44–51], vitamin C (2 papers) [52, 53], vitamin D (6 papers) [54–59], vitamin E (6 papers) [33, 60–62, 64, 83], vitamin K (3 papers) [63, 65, 66], and multiple vitamins (5 papers) [29, 67–70].

### Effect of mineral supplements on breast milk composition

In total, three minerals including zinc (3 papers), iron (4 papers) and selenium (6 papers) were reviewed. The summary of the effect of mineral supplements on breast milk content is presented in Tables 8, 9 and 10. Some findings are mentioned here, briefly.

#### Zinc

Two studies showed that zinc supplementation for lactating women positively influenced breast milk zinc levels [15, 72] and maternal body stores [72]. However, another study found that the milk zinc level decreased significantly for all lactating women, and there was no significant difference in the rate of declining zinc levels between women who started supplementation during lactation and those who were not supplemented [71].

#### Iron

Most studies found that iron supplementation did not significantly change iron levels of milk [80–82]. However, iron supplementation increased the total iron ligands in breast milk, measured by total iron-binding capacity and increased the proportion of lactoferrin in total protein secreted [81]. Breast milk lactoferrin levels seemed to be higher among supplemented women [81].

#### Selenium

Most studies showed that selenium supplementation increased breast milk selenium levels [73, 74, 76–78]; however, a recent study found that selenite supplementation was not related to a change of plasma or breast milk selenium concentrations [75].

### Effect of vitamin supplements on breast milk composition

In total, six vitamins including vitamin A, B, D, C, E and K in addition to multi-vitamin supplements were reviewed. The summary about the effect of vitamin supplements on breast milk content is presented in Tables 1, 2, 3, 4, 5, 6 and 7. Here, we mention some findings in brief.

#### Vitamin A

In total, 26 studies investigated the effect of vitamin A or its different supplementary forms including retinol, red palm oil (rich in pro-vitamin A), retinylpalmitate, β-carotene, and retinol palmitate on breast milk content. Vitamin A supplementation resulted in significantly increased retinol content, or α and β-carotene concentrations of breast milk in most studies [18–25, 27, 30, 31, 33, 35–39, 41, 42]. However, some studies found no association of vitamin A supplementation on breast milk composition [26, 28, 32, 34, 43].

#### Vitamin D

Vitamin D supplementation increased 25-hydroxy-D levels of breast milk [54–56, 58, 59], but in a recent study no significant incremental change was observed in 25 (OH) D levels of breast milk, however, change in 25 (OH) D levels in breast milk in the vitamin D supplemented group was significantly different from that of the placebo group [57].

#### Vitamin B

Dietary supplementation with vitamin B group, including B1 [47], B2 [47], B6 [45] and B12 [46, 49] was associated with increased levels of these vitamins in breast milk, however, no significant differences were observed for thiamin [48], and B12 [48] in some studies.

#### Vitamin C

Vitamin C supplementation resulted in a significantly higher ascorbic acid level in human milk [53], however in another study there was no significant change in maternal milk after vitamin C supplementation [52].

#### Vitamin K

Vitamin K supplemented groups had significantly higher vitamin K (phylloqinone) milk concentrations in most studies [63, 65, 66].

#### Vitamin E

Breast milk α-tocopherol levels increased after supplementation in the intervention group [62, 64, 83], however, no significant time-dependent changes were observed in breast milk [61]. Maternal supplementation with 400 international units of RRR, α, tocopherol increased vitamin E concentrations of the colostrum and transitional milk [60, 64, 83] but not of the mature milk [83].

#### Multiple vitamins

Significant increases were observed in the retinol and α-tocopherol levels of the colostrum among women who received vitamin A [68]. β-carotene supplementation did not significantly change the milk β-carotene concentrations, other carotenoids, retinol or tocopherol [29, 67] and retinol [67]. However, it is also reported that β-carotene supplementation might increase the mean β-carotene content of milk [67]. Another study showed that daily vitamin A and β-carotene supplementation during pregnancy and lactation resulted in increased retinol, β-carotene and α-carotene content of human milk at all time points during the first year postpartum [70]. However, supplementation with multi-vitamin (thiamin, riboflavin, vitamin B6, niacin, vitamin B12, vitamin C and E) was not associated with changes of β-carotene content, but it significantly decreased the γ- tocopherol levels of human milk at all times during lactation, and also reduced the retinol levels at delivery [70].

#### Previous systematic reviews

Based on our search in the mentioned databases, we found no comprehensive study quantifying the effects of dietary supplements including vitamins and minerals on breast milk composition. We found few systematic reviews on the effects of vitamin D [84], vitamin A [85], and vitamin K supplements on breast milk composition [86]. Additionally, two studies examined the effects of maternal nutrition and diet on breast milk composition, but not the effects of supplements on breast milk composition [2, 13].

#### Risk of bias of the included studies

According to Fig. 1, we found that the most common bias was related to random sequence generation and blinding. Also, allocation concealment was the most unclear risk of bias. The least bias included attrition bias and reporting bias.

## Discussion

This study systematically reviewed the effect of mineral and vitamin supplementation on breast milk composition. Different randomized controlled trials revealed the possible effect of maternal supplementation on breast milk content.

Breast milk of healthy, well-nourished mothers contains almost all the necessary nutrients for an infant’s growth and development. Association of maternal dietary supplements and milk composition might reflect the nutrient metabolism, since many ingredients of human milk are derived from maternal blood. In addition, understanding the effect of maternal nutrient supplementary intake on breast milk composition seems important, because almost all pregnant and lactating mothers receive supplements.

Previous studies have mostly focused on the effect of single vitamin or mineral supplementation on breast milk, while there is no comprehensive review on the effect of various vitamin and/or mineral supplementation during pregnancy and lactation and its impact on maternal milk composition.

### Vitamin A supplementation and breast milk

Randomized controlled trials evaluating the effect of postpartum maternal vitamin A supplementation indicated a significant improvement in maternal serum retinol, breast milk retinol and vitamin A liver stores, after single dose of vitamin A supplementation.

Vitamin A supplementation might be given in different formulations including vitamin A, measured in retinol units (IU) of retinylpalmitate, water miscible formulation or β-carotene. Synthetic β-carotene supplements resulted in improved breast milk vitamin A content, compared with dietary intake of β-carotene [87]. There is controversy regarding the duration of vitamin A supplementation, possibly due to the different breast milk collection methods. It has been suggested that although vitamin A supplementation did not show any adverse side effects, but this might not apply for women and infants from well-nourished populations [87].

Type of interventions were different between studies, including maternal vitamin A supplementation (β-carotene or retinylpalmitate or water miscible formulation) alone or in combination with other micro-nutrients (iron, folic acid, vitamin E) in comparison with placebo, no intervention, other micro-nutrient or a low dose of vitamin A [87]. In addition, co-existing vitamin A, zinc and iron deficiencies are common nutritional problems and evidence suggests that zinc status affects some aspects of vitamin A metabolism such as absorption, transportation and its usage [87].

It has been suggested that the baseline vitamin A status of breast milk might affect the results of supplementation studies [87]. Continued exclusive breastfeeding for 6 months indicated a greater cumulative need for vitamin A compared to mothers who give only 1, 2 or 3 breastfeeds per day. In addition, the follow up pattern of subjects with initial normal or high values of vitamin A is different from those with already low values at starting time [87]. Moreover, some studies did not clarify the techniques used for maternal milk collection or which breast was used, and time of milk collection was also not considered. Some studies suggest that it was not possible to distinguish between full-breast sample collection and on-demand collection [87].

As vitamin A is fat-soluble and carried in lipid phase, the variability of fat content of milk needs to be considered. This might also result in sampling errors due to non-standardized collection methods. This error could also be explained by the content of the breast from which the sample collection occurred; fuller breast usually have lower fat content [87, 88]. Moreover, fat content of milk which affects vitamin A concentrations is related to the time of day that samples are taken. Consistently, previous studies indicated greater agreement for lipid content in maternal milk collected between 6 AM and 8 AM of fasting women and greater variation in breast milk collected between 12 noon and 2 PM, and between 4 PM and 6 PM. This may explain differences between study findings without appropriate homogenization of procedures [89].

### Vitamin D supplementation and breast milk

The vitamin D content of human milk is completely variable and might be affected by season, maternal dietary intake of vitamin D, and ethnicity. Most previous studies have demonstrated that maternal vitamin D supplementation increased vitamin D content of human milk [54–56, 58, 59], but a recent study found no significant changes in breast milk 25 (OH) D levels [57]. It seems that as mothers provided milk samples at different time points including months 1, 2, 3 and 4 of lactation, this could have affected the study results. Also, recent evidence has shown that there is a possibility of correlation between some minerals and levels of vitamin D [90–92].

### Thiamin and riboflavin supplementation and breast milk

The main form of thiamin in milk is thiamin-monophosphate (TMP) with some free thiamin [93, 94], while flavin adenine dinucleotide (FAD) is the main source of riboflavin, in addition to free riboflavin and few amounts of other flavin compounds [95]. However, little is known about mechanisms related to the transport of vitamins B1 and B2 to breast milk or possible alterations of vitamer uptake due to supplementation. Previous studies reported that maternal thiamin supplementation resulted in higher thiamin content of breast milk [96], however, it seems that thiamin is transferred into breast milk in limited amounts [96]. Previous research in the US indicated no effect of thiamin supplementation on breast milk of well-nourished women [97, 98]. In addition, breast milk collected at 10 weeks will have considerably lower amounts of total thiamin compared to milk collected at 6 weeks [99].

The amounts of phosphorylated vitamers might either decrease or not change over time, therefore possible phosphorylation of thiamin, hydrolysis of thiamin pyrophosphate (TPP) or secretion of thiamin monophosphate (TMP) might be up-regulated in the early phase of lactation [47]. Among all vitamers of thiamin in human milk, only free thiamin levels increased during lactation which suggests an active transport of this vitamer to mammary glands. The major vitamer, TMP, might transport actively into the milk, but could also be found as a phosphorylation process of free thiamin or hydrolysis of TPP. Transportation, phosphorylation or hydrolysis processes might be up-regulated in the early stages of lactation.

Riboflavin supplementation is associated with higher levels of vitamin B2 in maternal milk [44, 96]. Free riboflavin is generally used in supplements, thus a greater increase in this vitamer is expected which suggests its efficient absorption and transport to breast milk, rather than conversion to its co-enzymatic forms prior to secretion. Supplementation might increase free riboflavin content of maternal milk, but not necessarily FAD levels, suggesting a favorable secretion of its free forms to breast milk [47]. More research is needed for a better understanding of mechanisms for vitamin secretion to maternal milk, factors associated with vitamer conversion in the mammary glands and the role of vitamers.

### Vitamin C supplementation and breast milk

Little is known about the impact of increased intake of vitamin C on human milk. Previous reports demonstrated that increased intake of vitamin C in women with low maternal vitamin C content at baseline, might increase vitamin C levels of human milk [52, 53, 100]. In a previous study, a relatively high dose of vitamin C caused a modest response among European women compared to a 3-fold increase in mean human milk vitamin C levels of African women [53]. It has been suggested that there is an upper limit for ascorbic acid secreted by the mammary glands, possibly due to the saturation of the gland with vitamin C [52, 100], however, the mechanism related to the regulation of ascorbic acid saturation and secretion by the glands are not completely understood. Differences between study findings suggest that there is significant variation in breast milk ascorbic acid content of individuals living in different regions. Moreover, milk samples were collected at different times, and the amount of milk expressed at each sampling varied. Lack of access to modern analytic techniques such as HPLC was another possible explanation; simple methods such as titration measures only reduced vitamin C (ascorbic acid) and not the total ascorbic acid. Based on previous studies, the dehydro-ascorbic acid level of human milk is lower than its reduced ascorbic content [53]. Dietary intake data was not collected in some studies, and urinary excretion of ascorbic acid was not monitored, as well [52, 53, 100].

### Vitamin E supplementation and breast milk

Studies on the effect of maternal vitamin E supplementation on breast milk are scarce and inconclusive; some reported a correlation between supplementation and breast milk vitamin E levels and some did not [60, 101, 102]. Maternal supplementation with R, R, R, α-tocopherol increased vitamin E levels of colostrum and transitional milk, but not the vitamin E of mature milk [102].

The positive effect of vitamin E supplementation on colostrum and transitional milk might be explained by increased synthesis of fatty acids by the mammary gland in the first few days after childbirth [103], due to the role of vitamin E in lipid metabolism. The mechanisms responsible for vitamin E transfer into breast milk have not been completely clarified, but it seems that α-tocopherol reaches the milk via LDL receptors [102, 104].

Previous studies indicated that vitamin E transportation to breast milk through independent and distinct mechanisms and limited by receptors, because maternal serum α-tocopherol content is not related to colostrum α-tocopherol levels [105]. Michaelis-Mentenkinetics is another hypothesis for α-tocopherol transfer to breast milk. This could transport vitamin E to the mammary gland regardless of its plasma levels [106, 107].

Some limitations of previous studies are as follows; reduced number of initial participants, and loss to follow up for analysis of α-tocopherol in mature milk. In addition, different dietary habits might explain differences between study groups [102]. The results provide evidence on the importance of maternal vitamin E supplementation, especially among women with preterm infants, to meet infant vitamin E requirements and protect them against oxidative stress [102, 108].

However, studies suggest that a single mega dose of 400 IU vitamin E seems not to be enough to increase vitamin E content of breast milk for a prolonged time [102].

### Β-carotene supplementation and breast milk

Information is lacking regarding changes in milk carotenoid content of healthy, well-nourished women in the first month of lactation. Role of carotenoids in breast milk is not completely understood. It has been suggested that high levels of carotenoids transferred to infants during the first few days of lactation could correct abnormally low levels of β-carotene in neonates [109]. Other studies have indicated that β-carotene supplementation of lactating mothers could increase the β-carotene content of breast milk [67, 110, 111].

Lack of increase in milk β-carotene content suggests that transitional milk might be saturated with β-carotene. The higher milk lutein levels and subsequent decrease in plasma lutein, suggests that lutein metabolism might be changed in early lactation [29]. The lack of effect of β-carotene supplements on retinol, α-tocopherol and other carotenoid levels of milk are consistent with previous reports [29, 67].

Different results might be explained by different techniques used to measure carotenoid content; before development of HPLC, separation methods reported only total carotenoid content [29], while HPLC technique provided more details on each specific carotenoid level. Moreover, interpretation was limited to small sample size, lack of maternal dietary intake data or lack of milk fat content as a variable. It has also been suggested to consider milk fat variations in milk carotenoid analysis [29, 33, 67, 112].

### Limitation and strengths

Because of large heterogeneity between studies, we could not conduct a meta-analysis; however, this comprehensive review provides sufficient information on the effect of maternal vitamin and/or mineral supplementation breast milk composition.

Other underlying factors including significant individual variation in diet, role of dietary intake data and analysis, over and/or underestimation of dietary intake, milk sample collection method, different dose and forms of supplementations (natural vs. synthetic), different techniques for nutrient measurements, race and/or ethnicity, postpartum milk sampling, time of milk sampling, baseline nutrient level of breast milk, and duration of breastfeeding, might partly explained the large variation between studies. Maternal baseline nutritional status might be considered prior to supplementation. In addition, some methodological differences between studies, including not using random allocation and/or blinding as an important part of a clinical trial, might affect study results.

### Limitations of the Jadad score

Considering three criteria (randomization, double-blinding, and a description of dropouts), the Jadad scale, is a common tool used to summarize quality measures of randomized controlled trials (RCTs). However, the Jadad scale has its limitations [113, 114]. First, the double blinding criterion is usually reported in around 10 to 20% of studies. Moreover, although the double blinding criterion accounts for 40% of the Jadad score, it is suggested that many trials involve devices, physical training, surgery, or other interventions e.g. exercise training for which double blinding is either impractical or impossible. In addition, the Jadad score does not assess the appropriateness of the data analysis, or allocation concealment, or of intention to treat, among other glaring deficiencies [115, 116].

## Conclusion

In conclusion, studies with different designs, e.g. not using random allocation and/or blinding, found that maternal dietary vitamin and/or mineral supplementation, particularly fat soluble vitamins, vitamins B1, B2 and C were reflected in breast milk composition. Moreover, vitamin supplements had greater effects on the breast milk composition compared to minerals. Higher dose of supplements showed more effects, and they were reflected in colostrum more than in mature milk. No difference was found between amega dose and a single dose administration of minerals.

## Data Availability

The datasets used and/or analyzed during the current study are available from the corresponding author on reasonable request.
